# Six-year outcome in subjects diagnosed with attention-deficit/hyperactivity disorder as adults

**DOI:** 10.1007/s00406-017-0850-6

**Published:** 2017-11-15

**Authors:** Dan Edvinsson, Lisa Ekselius

**Affiliations:** Department of Neuroscience, Psychiatry, Uppsala University, Uppsala University Hospital, Uppsala, Sweden

**Keywords:** ADHD, Adult, Outcome, Response, Pharmacotherapy

## Abstract

There are very few studies on the long-term outcome in subjects diagnosed with ADHD as adults. The objective of the present study was to assess this and relate the outcome to whether there was current medication or not and to other potential predictors of favourable outcome. A prospective clinical cohort of adults diagnosed with ADHD according to DSM-IV criteria was followed-up on an average of 6 years after first evaluation (*n* = 124; mean age 42 years, 51% males). ADHD symptom trajectories were assessed as well as medication, global functioning, disability, health-related quality of life, and alcohol and drug consumption at follow-up. Ninety percent of those diagnosed were initially treated pharmacologically and half of them discontinued treatment. One-third reported remission, defined as not fulfilling any ADHD subtype and a GAF-value last year ≥ 70, which was not affected by comorbidity at baseline. Current medication was not associated with remission. Subjects evaluated and first diagnosed with ADHD as adults are functionally improved at follow-up 6 years later despite a high percentage of psychiatric comorbidity at baseline. Half dropped out of medication, and there was no difference in ADHD remission between subjects with on-going medication at follow-up or subjects without medication, although current medication was related to a higher degree of self-reported global improvement.

## Introduction

A diagnosis of attention-deficit/hyperactivity disorder (ADHD) requires an onset of symptoms in early life and is estimated to affect about 5% of children [1]. Although symptoms decline over time, a high level of persistent functional loss may remain into adulthood [2]. In one study, 22% of subjects diagnosed with ADHD at the mean age of 8 years fulfilled criteria for ADHD at the age of 41 years [3], and in another study ADHD persisted into adulthood at 29% [4]. An estimate of the prevalence of ADHD in adulthood points to figures of 2.5–4.9% [5, 6], and with a high level of other psychiatric comorbidity [7–9]. Accordingly, adults with ADHD are overrepresented in a variety of settings and receive a wide range of treatment and measures within psychiatric health care and in other settings [10].

A subgroup of persons diagnosed with ADHD is first diagnosed in adulthood. This group is in many respects different from that diagnosed in childhood [11]. These differences have been summarized by Karam et al. [12] as (i) different type of referral and source of information; (ii) a higher prevalence in boys than in girls in childhood, but no difference in adults; (iii) an age-dependent decline of symptoms in child ADHD which might be less relevant during adulthood; (iv) the main complaint in childhood is hyperactivity/impulsivity, while inattention and executive dysfunction dominate in adults. (v) The profile of comorbidities differs with a dominance of disruptive behaviours in children, and a more complex adult profile, which is largely influenced by adolescent- and adult-onset psychiatric disorders, and finally (vi) the prevalence figures are puzzling as the adult prevalence is almost as high as the paediatric prevalence in spite of the fact that only a fraction of children with ADHD maintain a full diagnosis as adults. Taken together, all this suggest that studies on adult ADHD in those diagnosed as children cannot be uncritically transferred to those first diagnosed in adulthood. In fact, there is a large knowledge gap with respect to the long-term development of ADHD symptomatology over time in those subjects first diagnosed in adulthood. Furthermore, recent studies even propose that childhood onset and adult-onset ADHD may be distinct syndromes [13], or at least that there is an unexplained heterogeneity in the adult ADHD population [11].

The prevailing treatment strategy in ADHD is based on central stimulants. An abundance of studies have shown that such medication in children and adolescents is successful with respect to symptom reduction [10]. Similarly, a number of meta-analyses have suggested that central stimulants are effective in decreasing ADHD symptoms on a short-term basis also in adults [14, 15], and a single study did show effectiveness after 24 weeks of treatment in adults [16]. The response to treatment is, however, not predictable, due to a considerable, and not yet fully understood variability [17]. In general, however, evidence for long-term effectiveness remains elusive [18, 19], even if positive outcome and safety of treatment up to a year have been presented [20–22]. On the other hand, it has recently been argued that the clinical drug trials conducted in ADHD so far are of low quality and give little support for clinical decision making [23]. Nevertheless, a considerable fraction discontinue their medication, above all due to the perception that the medication is not effective or due to adverse effects [24].

As the aim of treatment is to achieve long-term symptom reduction, studies that report outcome after years of treatment are necessary. In the present study a well-defined cohort of subjects first diagnosed with ADHD as adults was assessed with respect to long-term outcome. This was also related to whether there was current medication or not and to other potential predictors of favourable outcome.

## Methods

### Subjects and design

**Fig. 1:**
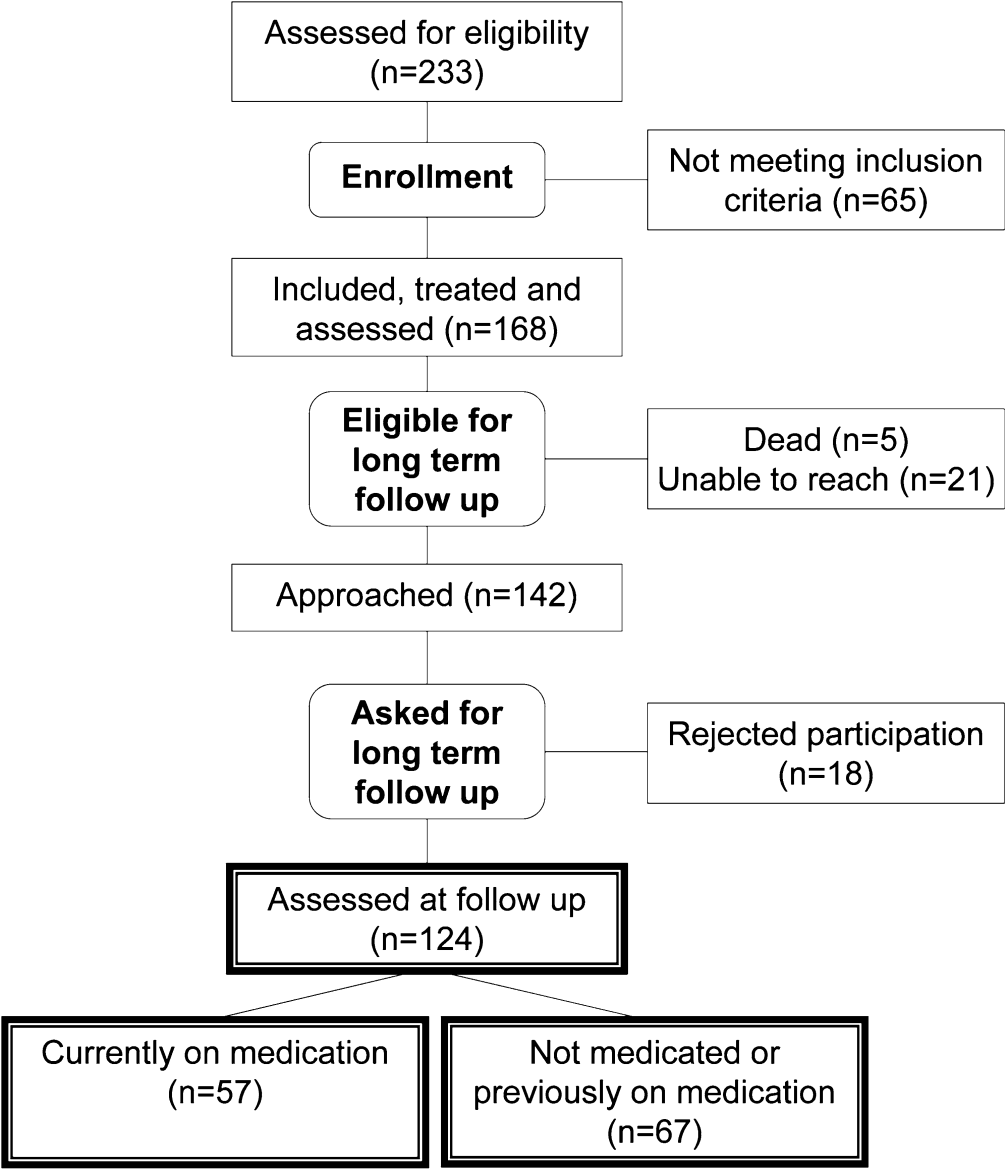
Diagram showing the flow of subjects through each stage of the study according to the CONSORT statement (http://www.consort-statement.org/)

In 2002, a special out-patient clinic for referral of adult subjects with suspected diagnoses of ADHD, autism spectrum disorders and Tourette syndrome was opened at Uppsala University Hospital. It was powered with a multidisciplinary team with senior psychiatrists, clinical psychologists, social workers and occupational therapists. The work was built on a stable structural diagnostic routine to enable follow-up for quality assurance and research purposes. Out of 233 subjects who were consecutively referred to a team led by senior psychiatrist D.E. between April 2002 and October 2010, 168 (78 women and 90 men) fulfilled diagnostic criteria of adult ADHD and were included in this study. See also Fig. 1.

The current follow-up was conducted between March and August 2013. Written informed consent was obtained from all included subjects. A questionnaire was distributed and later supplemented by an interview performed by a research nurse with extensive experience of handling subjects with ADHD. The study was approved by the Uppsala Regional Ethics and performed in accordance with the ethical standards laid down in the 1964 Declaration of Helsinki and its later amendments.

### Baseline assessment and treatment regimen

All included subjects were originally evaluated by a multidisciplinary team and diagnosed by the senior psychiatrist. The diagnostic procedure aimed at establishing best estimate diagnoses [25]. As reported in detail previously [9] ADHD symptomatology was assessed by a semi-structured interview based on the 18 DSM-IV criteria [26] and very similar to the SNAP-IV-18 [27]. In brief, answers were graded from 0 to 3 corresponding to symptom frequency and impairment: ‘never’, ‘sometimes’, ‘often’ and ‘very often’. An answer of ‘often’ or ‘very often’ was considered to represent a fulfilled DSM-IV criterion. The sum was presented as an ADHD-score, with a maximum value of 54. For the majority of subjects, childhood ADHD symptomatology could be confirmed by parents, other relatives, or childhood patient records. In 5 of the 124 included subjects diagnosis was based on self-reported childhood symptoms only, and two had diagnoses equivalent to ADHD in their childhood clinical records.

Current psychiatric comorbidity was evaluated by the Structured Clinical Interview for DSM-IV axis I disorders (SCID I) [28]. Criteria for DSM-IV axis II disorders were initially assessed by means of the DSM-IV and ICD-10 personality interview (DIP-I) [29], and after 2006 by the structured clinical interview for DSM-IV axis II (SCID-II) [30], which is very similar to the DIP-I, and was utilized for half of the included subjects. Figures for axis II disorders are reported based on the specific criteria only. The diagnostic interview for social and communication disorders [31], and the autism diagnostic interview-revised [32] were conducted by senior clinical psychologists for evaluations of suspected autism spectrum disorders.

All subjects were subject to physical examination including basic neurologic status, as well as routine laboratory testing including screening for illegal drugs.

The treatment protocol was individualised for each patient and based on a multimodal approach containing both pharmacological and a non-pharmacological measures.

### Follow up

Current symptoms and treatment history were assessed on average of 77 months after baseline and performed in two steps.

First, questionnaires were sent to the included subjects with questions about current symptoms of ADHD using a self-report version of the semi-structured interview utilized at baseline. Current functioning was assessed by a self-report version of the global assessment of functioning scale (GAF) [33]. Potential improvement since baseline was measured by a self-report version of the clinical global improvement scale (CGI-I), scoring 1–7, corresponding to answers “very much improved” to “very much worse” [34]. Disability and impairment were investigated by the Sheehan disability scale (SDS) [35] covering three domains: work/school, social life and family life/home responsibilities. SDS has recently been psychometrically evaluated in adult ADHD [36]. Health-related quality of life (HRQoL) was measured by EQ-5D and EQ-VAS [37]. Alcohol and drug consumption was assessed by the alcohol use disorders identification test (AUDIT) [38] and drug use disorders identification test (DUDIT) [39]. Twenty-nine participants were given support to fill in the questionnaires, 16 obtained telephone support and 13 were supported in their homes or during a visit to the clinic.

Second, after return of the questionnaires there was a telephone interview covering demographics as well as details of their past and current medical condition. The interview mainly functioned to validate that the patients have understood the questionnaires, and to give practical support to fill in the questionnaires. Twenty-nine participants were given such support.

Subjects’ own information regarding previous medication periods was validated against data in their patient files if available. In case of contradictory data, we choose to rely on patient file data. Slightly less than half of the subjects, 57 subjects; 46%, were on current medication, while 67 subjects were not. Mono-therapy was reported by all but three participants; methylphenidate in 46 subjects and amphetamine in eight subjects. Three subjects (5%) were treated with methylphenidate and atomoxetine in combination. A vast majority (84%) reported adherence to treatment on a daily basis. Fifty-five of those medicated had discontinued their medication and 12 (10%) were never treated. The 55 who had discontinued medication had been free of drugs for 41 ± 27 months; range 1–108, and 49 out of those had been free of drugs for a year or more at follow-up. In only two subjects the reason for discontinuation was the person’s own perceived clinical improvement.

Non-pharmacological measures included psychoeducation (received by 41%), social and similar support (34%), vocational support (44%) and psychological therapy (55%), either in a group or individualised. Any non-pharmacological intervention was given to 80% of the subjects.

### Primary outcome measure and statistics

The primary outcome measure was “remission”, which was defined as not fulfilling any ADHD subtype and a GAF-value of ≥ 70 during the last year.

Dichotomous data were analysed using Chi-square test statistics or Fisher’s exact test when applicable. Kolmogorov–Smirnov’s test was used to test for normality. When appropriate Mann–Whitney *U* test was used for analysis of group differences; otherwise the *t* test was performed for continuous data.

The strategy was to assess potential predictors of remission in stepwise binary logistic regression analyses, where variables which were significant in simple regressions should be included in a final model. Values are given as mean ± SD, or median with range within brackets. All statistical analyses were performed using IBM SPSS Statistics, version 23.

## Results

Out of the 168 eligible subjects, 124 (74%) could be included for follow-up (Fig. 1). Two subjects returned their questionnaires but did not participate in the subsequent interview, whereas one person did the opposite. A full dataset was consequently available for 122 and 123 subjects, respectively.

There was no baseline difference between the 124 participants and the 44 non-participants with respect to age, sex, ADHD subtype, ADHD-score, axis I or axis II comorbidity, autism spectrum disorders or education level (data not shown). However, participants more often lived alone (*p* < 0.001), worked full/part time or were studying (*p* < 0.05).

Table 1 shows the characteristics of the subjects at baseline and at follow-up.

**Table 1:** Key baseline and follow-up characteristics of subjects diagnosed with ADHD as adults

	Baseline	Follow-up
Age (years)	35 ± 9	42 ± 10
Follow-up time (time since evaluation; months)		77 ± 24 76 (31–133)^b^
Duration of medication (months)^a^		40 ± 39 39 (0–110)^b^
Gender [females/males; *n* (%)]	61/63 (49/51)	61/63 (49/51)
ADHD subtype^c^		
Predominantly inattentive	47 (38)	31 (25)
Combined	71 (58)	21 (17)
Predominantly hyperactive/impulsive	5 (4)	7 (6)
Do not fulfil criteria for ADHD at follow-up		64 (52)
ADHD score^d^	36.8 ± 7.8	25.5 ± 11.1
Remission; yes/no		41/82 (33/67)
Current psychiatric comorbidity	59/65 (48/52)	
Mood disorder (yes/no)	15/109 (12/88)	
Anxiety disorder (yes/no)	37/87 (30/70)	
Eating disorder (yes/no)	2/122 (2/98)	
Adjustment disorder (yes/no)	2/122 (2/98)	
Psychotic disorder (yes/no)	1/123 (1/99)	
Substance use disorder (yes/no)	6/118 (5/95)	
Autism spectrum (yes/no)	12/112 (10/90)	
Tourette syndrome (yes/no)	5/119 (4/96)	
Any personality disorder (yes/no)	52/72 (42/58)	
Number of fulfilled personality disorder criteria	8 ± 6	
Living conditions^e^		
Living alone	56 (46)	58 (48)
Living with partner	57 (47)	57 (47)
Living with parent/relative	5 (4)	0 (0)
Supported housing	4 (3)	7 (6)
Education^e^		
Incomplete compulsory school	7 (6)	2 (2)
Compulsory school	42 (34)	41 (33)
Sixth form	36 (30)	29 (24)
Incomplete university	20 (16)	27 (22)
University degree	17 (14)	23 (19)
Work status^e^		
Full time	36 (29)	41 (34)
Part time	12 (10)	31 (25)
Student	13 (11)	5 (4)
Unemployed	22 (18)	17 (14)
Sick leave/pension	39 (32)	28 (23)

Figures within brackets represent percent unless stated otherwise

^a^Methylphenidate (*n* = 46), amphetamine (*n* = 8) and methylphenidate and atomoxetine in combination (*n* = 3). The mean daily doses were 60 ± 32, 42 ± 9 mg, and for atomoxetine 38 ± 13 mg, respectively

^b^Median and range in brackets

^c^ *n* = 123

^d^ *n* = 119

^e^ *n* = 122

### Longitudinal assessment of ADHD symptomatology and response to medication

**Fig. 2:**
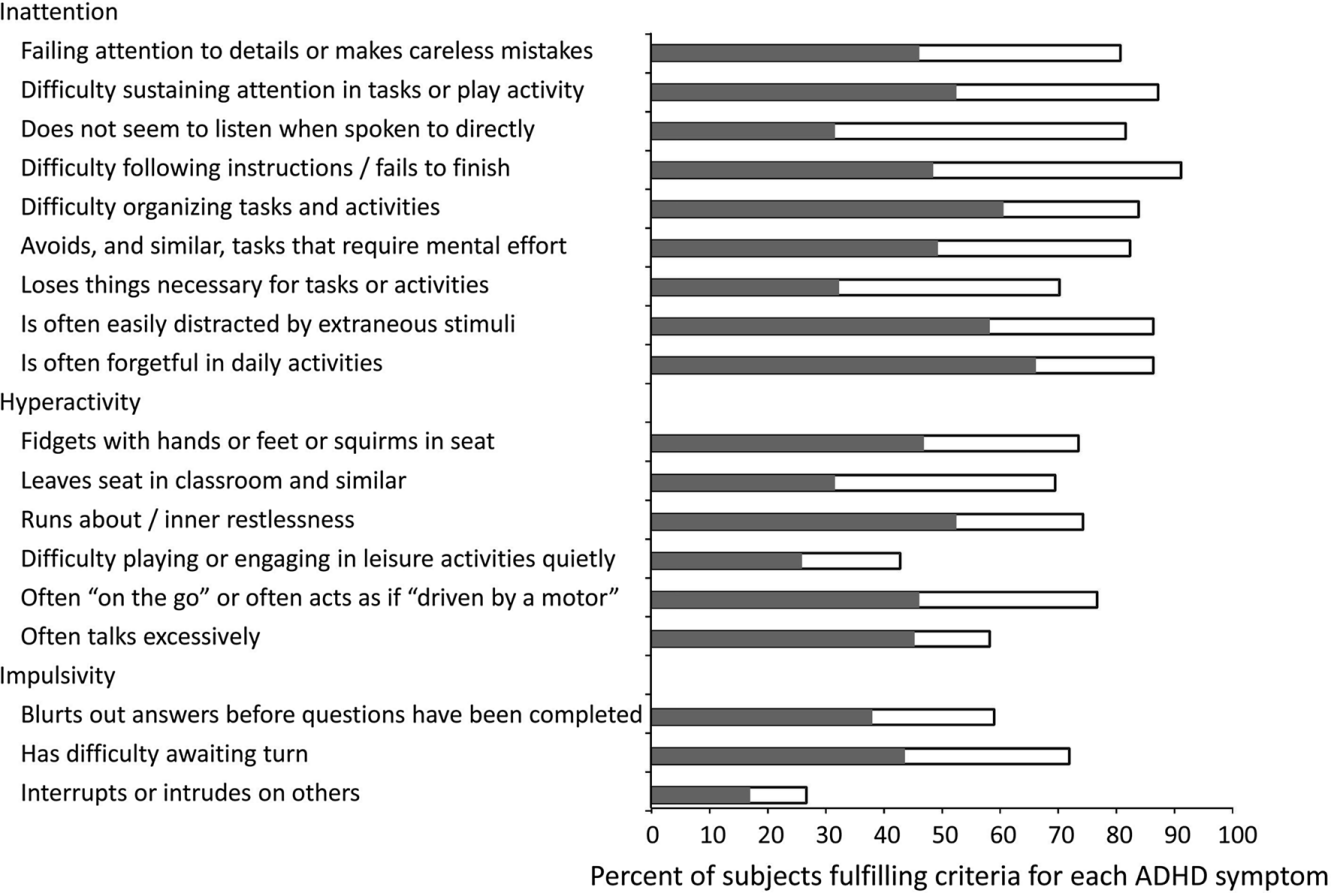
Percent of individuals fulfilling each DSM-IV ADHD symptom at baseline, open bar, and at follow-up, shaded bar

At follow-up, there was a decrease in mean ADHD score from 36.8 ± 7.8 to 25.5 ± 11.1 and 33% of the subjects, 41 out of 123, were in remission (Table 1). There was also a decrease in the number of subjects who fulfilled each of the 18 different DSM-IV ADHD criteria, and which was similar for each of the criteria (Fig. 2). It was also noted that there was an apparent change in ADHD subtype over the observation period.

Those in remission reported similar ADHD scores at baseline as those who were not in remission. Furthermore, there was no difference in current medication between those groups; 51% vs. 43% (Table 2). Reciprocally, there was no difference in remission rate between subjects in the current medication group, 37%, and in those not currently medicated, 30% (Table 3). Those medicated reported similar ADHD scores as those without ongoing medication both at baseline and at follow-up. The change in ADHD score between the two time periods was also very similar. Finally, there were no significant differences between the current-treatment group and those not medicated with respect to age, sex, or axis I or axis II comorbidity at baseline (data not shown).

**Table 2:** Data for the subjects that responded to the enquiry at follow-up divided by state of remission

	Remission (*n* = 41)	Not remission (*n* = 82)	*p*
Duration of medication (months)	51 ± 31^b^	41 ± 30^c^	< 0.001^a^
Duration of medication (percent of follow-up time)	65 ± 35^b^	55 ± 35^c^	< 0.001^a^
ADHD score at baseline	35.8 ± 7.7^d^	37.2 ± 7.8^e^	0.41^a^
ADHD score at follow-up	15.6 ± 7.5	30.5 ± 8.8	< 0.001^a^
ADHD score; change	20.7 ± 9.9^d^	6.8 ± 10.2^e^	< 0.001^a^
Current medication	21 (51%)^b^	35 (43%)^c^	0.44
CGI-improvement	2.2 ± 1.3	3.2 ± 1.7	< 0.001^a^
GAF last year	84 ± 10	61 ± 14	< 0.001^a^
GAF last 2 weeks	82 ± 17	62 ± 16	< 0.001^a^
Sheehan disability scale (SDS)			
Work/school	1.7 ± 3.1	4.5 ± 3.8	< 0.001^a^
Social life	1.7 ± 3.4	4.5 ± 3.5	< 0.001^a^
Family life/home responsibilities	1.5 ± 2.7	4.0 ± 3.4	< 0.001^a^
EQ-5D index	0.82 ± 0.19	0.55 ± 0.33	< 0.001^a^
EQ-5D VAS (visual analogue scale)	78 ± 14	57 ± 21	< 0.001^a^
AUDIT score	3.5 ± 3.8	4.0 ± 4.4	0.67^a^
DUDIT score	0.2 ± 0.7	1.3 ± 4.3	0.12^a^

^a^Mann–Whitney

^b^ *n* = 35

^c^ *n* = 76

^d^ *n* = 38

^e^ *n* = 80

**Table 3:** Data for the subjects that responded to the enquiry at follow-up divided by state of medication

	Current medication		*p*
	Yes (*n* = 56)	No (*n* = 67)	
Duration of medication (months)	63 ± 24; median 62 (10–110)	20 ± 22; median 10 (0–75)	< 0.001^a^
Duration of medication (percent of follow-up time)	84 ± 19; median 88 (16–100)	19 ± 27; median 15 (0–93)	< 0.001^a^
Medication free time before follow-up assessment (months)^b^		41 ± 27; median 40 (1–108)	
ADHD score at baseline	36.5 ± 8.6^c^	37.2 ± 7.2^d^	0.67^a^
ADHD score at follow-up	24.5 ± 11.0^c^	26.4 ± 10.9	0.67^a^
ADHD score; change	11.8 ± 11.9^c^	10.8 ± 12.1^d^	0.57^a^
Do not fulfil criteria for ADHD at follow-up	31 (55%)	33 (49%)	0.50
Remission	21 (37%)^c^	20 (30%)	0.44
CGI-improvement	2.4 ± 1.5^c^	3.3 ± 1.6	< 0.001^a^
GAF last year	69 ± 16^c^	68 ± 17	0.90^a^
GAF last 2 weeks	69 ± 18^c^	68 ± 20	0.86^a^
Sheehan disability scale (SDS)			
Work/school	3.2 ± 3.7^c^	3.8 ± 3.8	0.41^a^
Social life	3.4 ± 3.6^c^	3.7 ± 3.8	0.74^a^
Family life/home responsibilities	2.8 ± 3.3^c^	3.4 ± 3.4	0.34^a^
EQ-5D index	0.68 ± 0.30^c^	0.60 ± 0.33	0.17^a^
EQ-5D VAS (visual analogue scale)	67 ± 19^c^	62 ± 24	0.19^a^
AUDIT score	4.1 ± 4.5^c^	3.6 ± 4.0	0.50^a^
DUDIT score	1.5 ± 5.1^c^	0.4 ± 1.2	0.21^a^

^a^Mann–Whitney

^b^The 55 who had discontinued their treatment

^c^ *n* = 56

^d^ *n* = 64

### Cross-sectional measures of functioning and quality of life at follow-up

#### Remission vs. not remission

Those who were in remission reported significantly better global functioning, less disability, better quality of life, and also better clinical improvement, than those who were not in remission (Table 2). The global functioning at follow-up was only moderately affected with mean GAF scores close to 80 in those in remission and close to 60 in those who were not in remission, although with variations (Table 2). The SDS suggested in general only mild–moderate impact on the domains work/school, social life, and for family life/home responsibilities in those that were in remission, although with a large difference between individuals (Table 3). Half, or more than half of those that were not in remission, however, reported a score of 5 or more in the three domains, suggesting significant impairment (50, 55, 54% in those who were not in remission and 17, 15 and 15% in those who were in remission, for the three domains work/school, social life, and for family life/home responsibilities, respectively).

There was no difference in mean alcohol consumption, as measured by AUDIT scores and drug use as assessed by DUDIT scores. Scores above limits for harmful drinking [38] were, however, observed in 7% of those in remission versus 24% of those who were not (*p* = 0.03). Any use of drugs other than alcohol was reported in 7% of those in remission and 17% in those who were not; totally 14%. Risk scores for potential drug-related problems [39] was reported in 2% of those in remission versus 11% of those who were not; totally 8% (*p* = 0.16).

#### Current medication vs. no medication

Although those who had current medication did not report better on longitudinal assessments of their ADHD trajectories they reported significantly better on the CGI-I scale than those who had no medication (Table 3). There was, however, no difference in perceived quality of life measured by EQ-5D index and VAS, or in GAF and SDS scores.

### Prediction analysis

All baseline variables recorded in Table 1, plus treatment-related variables, were tested for possible inclusion in a regression model with remission as the dependent variable. None of these variables was, however, significantly related to remission.

## Discussion

The first main finding is that one-third of the subjects first diagnosed with ADHD as adults went into remission during an observation time of on average 6 years. This result relates well to findings in two recent similar studies. Karam et al. [12] described the result of a 7-year longitudinal study of adults with ADHD. With a response rate of 66%, approximately one-third did not maintain ADHD criteria at follow-up and 12% had full remission. Lensing et al. made a questionnaire survey in adults with ADHD who were approved for pharmacotherapy in Norway and obtained a response rate of 34% after a mean observation time of 4.5 years. They observed that 48% were below a cut-off level for ADHD [40]. This was similar to the proportion in the present study.

The second finding is that, despite a high percentage of psychiatric comorbidity at baseline, adult ADHD in general seems to have a favourable long-term outcome judged from the GAF scores at follow-up. These scores were similar to values previously reported from the general population [41]. Furthermore, the domain means on the SDS were below the cut-off for significant functional impairment also in those who were not in remission. The EQ-5D index, a proxy for HRQoL, was similar to population sample values [42] in those who were in remission, but lower in those who were not in remission. There was, however, a substantial variability between individuals with respect to these measures, which indicates that some of the subjects still perceived substantial suffering with respect to functioning, disability and HRQoL. Finally, the alcohol consumption, as measured by AUDIT, was similar to that in the Swedish general population [43]. On the other hand, DUDIT scores which reflected any use of drugs was reported in 14% overall, and in 17% of non-remitters, which is higher than the 3% (4.8% in men and 1.6% in women) in the Swedish general population [39].

The third main finding was that the remission rate and the ADHD scores were similar in those who were on current treatment with central stimulants and in those who were not treated. This observation adds to the present literature as there is currently no accepted evidence based on longitudinal assessments which suggests that there are long-term benefits from such medication in those first diagnosed with ADHD as adults [19]. Actually, it was recently reported that pharmacological treatment had no beneficial impact on either ADHD symptom severity or overall functioning after 6 years in individuals treated in late adolescence and young adulthood [44]. Moreover, Karam et al. [12] reported that continued medication was not related to remission. On the other hand, Lensing et al. [40] reported that a higher proportion was below a cut-off score for ADHD in the currently medicated group. Also, their quantitative ADHD scores differed similarly, as did also screen data for mental health and self-reported improvement.

The fourth main finding was that half of the subjects treated with central stimulants had discontinued treatment at follow-up which is similar to data reported from previous long-term studies [24, 40]. Actually high dropout figures is the rule in studies on adult ADHD, a problematic feature which has been well exploited previously [52]. Low adherence, including discontinuation, has been related to a suboptimal treatment effect [45].

Even though those who were currently on medication did not report better ADHD scores or Sheehan disability scores, they still reported higher improvement measured by CGI-I. This discrepancy may suggest that there are medication-related improvements which are not adequately reflected in the symptom-specific ADHD-screening profile but still sensed by the subjects. It is also possible that the subjects’ ratings contain a placebo-related mechanism in those who are compliant with the medication and pursue treatment over time. Finally, clinically not discerned individual differences may affect both a possible response to, and a tendency to discontinue medication [46, 47].

It is noteworthy that no baseline factors predicted outcome 6 years after being first diagnosed with ADHD in adulthood. This stands in contrast to previous studies which indicate that psychiatric comorbidity at baseline was related to poorer outcome after on average 4.5 years [40], and also that the presence of antisocial personality disorder was related to short duration of treatment [48]. The latter is in contrast to an observation that the presence of a comorbid disorder did not affect adherence to or persistence on stimulant medication after 24 weeks [49]. There are several possible explanations for such discrepant results. One such is related to the characteristics of the patient sample. Our outpatient clinic received patients whose problems were dominated by ADHD-like symptomatology, which may have led to an underrepresentation of patients where comorbidities like substance use disorders or affective disorders dominated the clinical picture. Another explanation could be that comorbidities are defined, registered and diagnosed differently in different studies. In the present study, comorbidities were defined as current DSM-IV disorders diagnosed in a full SCID interview at baseline.

An issue is that the primary outcome variable differs between studies. It has been recommended that clinical research in ADHD should use “remission” as the primary outcome [50], thus, the said approach was used in this study. This paper defined “remission” as not fulfilling any ADHD subtype and a GAF-value of 70 or higher during the last year. Nevertheless, we also calculated persistence of symptoms as done by Biederman et al. [51] in the form of full or subthreshold ADHD based on the DSM-IV diagnostic criteria, or failure to attain functional remission (GAF score ≤ 60). Irrespective of definition chosen there was no significant difference between those who were on current medication and those who were not (data not shown).

The clinical implications are that half of adults diagnosed with ADHD, and subsequently pharmacologically treated, had discontinued medication after a mean period of 6 years. On-going medication at follow-up was related to improvement of variables beyond core ADHD symptomatology such as global improvement and increased quality of life. As the study is small, although with a long follow-up time, it is difficult to draw detailed conclusions from subgroups in a trustworthy manner. In a broader context, the study support the notion that this group of patients constitutes a clinical challenge where each person requires individually designed support containing a multitude of approaches, and where medication is only one. The observation that half of the patients terminate medication after some time and where the termination is not related to improved symptoms of ADHD, points to a structural weakness in ability to motivate and support patients [52]. Awaiting reliable drug trials [23] on which to support scientifically based routines for long-term treatment, the only reasonable clinical approach is to search for best possible way to follow and monitor individual patients over a long time and to take measures to prevent discontinuation of treatment.

### Limitations

First, this study did not evaluate on-going psychiatric comorbidity at follow-up. There was, however, no difference in the number of comorbidities at baseline between those that remitted and those that did not, or in those that continued and those who discontinued their medication. Furthermore, in a prior study those in remission did not have more remission from comorbidities at follow-up than those being non-remitters [12]. Second, ADHD baseline data were obtained by an interview and follow-up in self-report form, although using the same questionnaire. This modality was chose of practical reasons and knowing that there was evidence that self-assessment of ADHD symptoms present with an acceptable validity and reliability; see, e.g., references [53, 54]. Likewise, a subject recall-bias over, e.g., adherence to treatment may be an issue over a period as long as 6 years [55]. On the other hand has been documented that adults can provide a reliable rating both of their own childhood and current symptoms in ADHD [56]. Third, differences in non-pharmacological treatment and/or supporting interventions since baseline are potential factors that could have biased the results. There were, however, no difference in the number of such interventions between those who continued and those who discontinued their medication and between those who were on remission and those who were not (data not shown). This observation must, however, be interpreted with caution due to the very low statistical power. Fourth, this cohort study was commenced at a time when initiation of treatment with stimulants was highly centralised to specialized centres, and when the awareness about the condition “adult ADHD” was low. This reasonably led to a selection bias. Consequently, results can a priori not be said to contradict those that are obtained in studies based on different modes of patient recruitment or follow-up times [24], or with a considerably higher attrition [40]. Fifth, this is a naturalistic study, and it is not random whether subjects ended up in the medicated or in the not-mediated group at follow-up. A faulty conclusion would be to avoid considering medication again in non-remitted subjects who have previously discontinued their pharmacological treatment. Finally, there is currently lack on sound information on whether any of the many scales used to assess ADHD is better than any other scales [57]. The scale scoring system used by us was constructed at a time when there was no widespread and internationally accepted scoring method based on all 18 DSM-IV criteria. It is very similar to the widely used adult ADHD self-report scale ASRS-1 which was published in 2005 [58], as is also the SNAP-IV-18 which, however, is phrased for children [27]. Consequently, there is no reason to suppose that the use of another similar scale would have generated different results.

### Strengths

This paper presents one of the longest naturalistic outcome studies of clinical subjects diagnosed with ADHD in adulthood. It is characterized by a very low attrition rate and almost three-fourth of the cohort was assessed after a mean of 6 years, which is substantially more than in previous prospective studies which present with dropout rates as high as 50–66% [24, 40]. All subjects were included consecutively after being diagnosed with ADHD and assessed by one senior psychiatrist. As subjects were first diagnosed and exclusively treated as adults there are no potential persisting effects of previous treatment in childhood.

## References

[1] Polanczyk GV, Salum GA, Sugaya LS, Caye A, Rohde LA (2015). Annual research review: a meta-analysis of the worldwide prevalence of mental disorders in children and adolescents. J Child Psychol Psychiatry.

[2] Faraone SV, Biederman J, Mick E (2006). The age-dependent decline of attention deficit hyperactivity disorder: a meta-analysis of follow-up studies. Psychol Med.

[3] Klein R, Mannuzza S, Olazagasti M, Roizen E, Hutchison J, Lashua E, Castellanos F (2012). Clinical and functional outcome of childhood attention-deficit/hyperactivity disorder 33 years later. Arch Gen Psychiatry.

[4] Barbaresi WJ, Colligan RC, Weaver AL, Voigt RG, Killian JM, Katusic SK (2013). Mortality, ADHD, and psychosocial adversity in adults with childhood ADHD: a prospective study. Pediatrics.

[5] Simon V, Czobor P, Balint S, Meszaros A, Bitter I (2009). Prevalence and correlates of adult attention-deficit hyperactivity disorder: meta-analysis. Br J Psychiatry.

[6] Ramos-Quiroga JA, Nasillo V, Fernandez-Aranda F, Casas M (2014). Addressing the lack of studies in attention-deficit/hyperactivity disorder in adults. Expert Rev Neurother.

[7] McGough JJ, Smalley SL, McCracken JT, Yang M, Del’Homme M, Lynn DE, Loo S (2005). Psychiatric comorbidity in adult attention deficit hyperactivity disorder: findings from multiplex families. Am J Psychiatry.

[8] Sobanski E, Bruggemann D, Alm B, Kern S, Deschner M, Schubert T, Philipsen A, Rietschel M (2007). Psychiatric comorbidity and functional impairment in a clinically referred sample of adults with attention-deficit/hyperactivity disorder (ADHD). Eur Arch Psychiatry Clin Neurosci.

[9] Edvinsson D, Lindstrom E, Bingefors K, Lewander T, Ekselius L (2013). Gender differences of axis I and II comorbidity in subjects diagnosed with attention-deficit hyperactivity disorder as adults. Acta Neuropsychiatr.

[10] Thapar A, Cooper M (2016). Attention deficit hyperactivity disorder. Lancet.

[11] Agnew-Blais JC, Polanczyk GV, Danese A, Wertz J, Moffitt TE, Arseneault L (2016). Evaluation of the persistence, remission, and emergence of attention-deficit/hyperactivity disorder in young adulthood. JAMA Psychiatry.

[12] Karam RG, Breda V, Picon FA, Rovaris DL, Victor MM, Salgado CA, Vitola ES, Silva KL, Guimaraes-da-Silva PO, Mota NR, Caye A, Belmonte-de-Abreu P, Rohde LA, Grevet EH, Bau CH (2015). Persistence and remission of ADHD during adulthood: a 7-year clinical follow-up study. Psychol Med.

[13] Faraone SV, Biederman J (2016). Can attention-deficit/hyperactivity disorder onset occur in adulthood?. JAMA Psychiatry.

[14] NICE (2008) Attention deficit hyperactivity disorder: diagnosis and management of ADHD in children, young people and adults. NICE clinical guideline 72. Last updated February 2016. National Institute for Health and Care Excellence. https://www.nice.org.uk/guidance/cg72. Accessed 17 Aug 2016

[15] Moriyama TS, Polanczyk GV, Terzi FS, Faria KM, Rohde LA (2013). Psychopharmacology and psychotherapy for the treatment of adults with ADHD-a systematic review of available meta-analyses. CNS Spectr.

[16] Rosler M, Fischer R, Ammer R, Ose C, Retz W (2009). A randomised, placebo-controlled, 24-week, study of low-dose extended-release methylphenidate in adults with attention-deficit/hyperactivity disorder. Eur Arch Psychiatry Clin Neurosci.

[17] Retz W, Retz-Junginger P (2014). Prediction of methylphenidate treatment outcome in adults with attention-deficit/hyperactivity disorder (ADHD). Eur Arch Psychiatry Clin Neurosci.

[18] Shaw M, Hodgkins P, Caci H, Young S, Kahle J, Woods AG, Arnold LE (2012). A systematic review and analysis of long-term outcomes in attention deficit hyperactivity disorder: effects of treatment and non-treatment. BMC Med.

[19] Hinshaw SP, Arnold LE, MTA Cooperative Group (2015). Attention-deficit hyperactivity disorder, multimodal treatment, and longitudinal outcome: evidence, paradox, and challenge. WIREs Cogn Sci.

[20] Weisler R, Young J, Mattingly G, Gao J, Squires L, Adler L (2009). Long-term safety and effectiveness of lisdexamfetamine dimesylate in adults with attention-deficit/hyperactivity disorder. CNS Spectr.

[21] Biederman J, Mick E, Surman C, Doyle R, Hammerness P, Kotarski M, Spencer T (2010). A randomized, 3-phase, 34-week, double-blind, long-term efficacy study of osmotic-release oral system-methylphenidate in adults with attention-deficit/hyperactivity disorder. J Clin Psychopharmacol.

[22] Huss M, Ginsberg Y, Tvedten T, Arngrim T, Philipsen A, Carter K, Chen CW, Kumar V (2014). Methylphenidate hydrochloride modified-release in adults with attention deficit hyperactivity disorder: a randomized double-blind placebo-controlled trial. Adv Ther.

[23] Boesen K, Saiz LC, Erviti J, Storebo OJ, Gluud C, Gotzsche PC, Jorgensen KJ (2017). The Cochrane Collaboration withdraws a review on methylphenidate for adults with attention deficit hyperactivity disorder. Evid Based Med.

[24] Bejerot S, Ryden EM, Arlinde CM (2010). 2-year outcome of treatment with central stimulant medication in adult attention-deficit/hyperactivity disorder: a prospective study. J Clin Psychiatry.

[25] Klein DN, Ouimette PC, Kelly HS, Ferro T, Riso LP (1994). Test-retest reliability of team consensus best-estimate diagnoses of axis I and II disorders in a family study. Am J Psychiatry.

[26] American Psychiatric Association (2000). Diagnostic and statistical manual of mental disorders: DSM-IV-TR.

[27] Swanson J (1992). School-based assessment and interventions for ADD students.

[28] First M, Spitzer RL, Gibbon M, Williams JBW (1996). Structured clinical interview for DSM-IV axis I disorders, clinical version (SCID-CV).

[29] Ottosson H, Bodlund O, Ekselius L, Grann M, von Knorring L, Kullgren G, Lindstrom E, Soderberg S (1998). DSM-IV and ICD-10 personality disorders: a comparison of a self-report questionnaire (DIP-Q) with a structured interview. Eur Psychiatry.

[30] First MB, Gibbon M, Spitzer RL, Williams JBW, Benjamin LS (1997). Structured clinical interview for DSM-IV axis II personality disorders (SCID-II).

[31] Leekam SR, Libby SJ, Wing L, Gould J, Taylor C (2002). The diagnostic interview for social and communication disorders: algorithms for ICD-10 childhood autism and Wing and Gould autistic spectrum disorder. J Child Psychol Psychiatry.

[32] Le Couteur A, Rutter M, Lord C, Rios P, Robertson S, Holdgrafer M, McLennan J (1989). Autism diagnostic interview: a standardized investigator-based instrument. J Autism Dev Disord.

[33] Ramirez A, Ekselius L, Ramklint M (2008). Axis V—global assessment of functioning scale (GAF), further evaluation of the self-report version. Eur Psychiatry.

[34] Guy W (1976). ECDEU assessment manual for psychpharmacology.

[35] Leon AC, Olfson M, Portera L, Farber L, Sheehan DV (1997). Assessing psychiatric impairment in primary care with the Sheehan disability scale. Int J Psychiatry Med.

[36] Coles T, Coon C, DeMuro C, McLeod L, Gnanasakthy A (2014). Psychometric evaluation of the Sheehan disability scale in adult patients with attention-deficit/hyperactivity disorder. Neuropsychiatr Dis Treat.

[37] The EuroQol Group (1990). EuroQol—a new facility for the measurement of health-related quality of life. Health Policy.

[38] Babor TF, Higgins-Biddle JC, Saunders JB, Monteiro MG (2001). AUDIT. The alcohol use disorders identification test. Guidelines for use in primary care.

[39] Berman AH, Bergman H, Palmstierna T, Schlyter F (2005). Evaluation of the drug use disorders identification test (DUDIT) in criminal justice and detoxification settings and in a Swedish population sample. Eur Addict Res.

[40] Lensing MB, Zeiner P, Sandvik L, Opjordsmoen S (2013). Four-year outcome in psychopharmacologically treated adults with attention-deficit/hyperactivity disorder: a questionnaire survey. J Clin Psychiatry.

[41] Edvinsson D, Bingefors K, Lindstrom E, Lewander T (2010). ADHD-related symptoms among adults in out-patient psychiatry and female prison inmates as compared with the general population. Ups J Med Sci.

[42] Burstrom K, Johannesson M, Diderichsen F (2001). Swedish population health-related quality of life results using the EQ-5D. Qual Life Res.

[43] Kallmen H, Wennberg P, Leifman H, Bergman H, Berman AH (2011). Alcohol habits in Sweden during 1997–2009 with particular focus on 2005 and 2009, assessed with the AUDIT: a repeated cross-sectional study. Eur Addict Res.

[44] van Lieshout M, Luman M, Twisk JW, van Ewijk H, Groenman AP, Thissen AJ, Faraone SV, Heslenfeld DJ, Hartman CA, Hoekstra PJ, Franke B, Buitelaar JK, Rommelse NN, Oosterlaan J (2016). A 6-year follow-up of a large European cohort of children with attention-deficit/hyperactivity disorder-combined subtype: outcomes in late adolescence and young adulthood. Eur Child Adolesc Psychiatry.

[45] Perwien A, Hall J, Swensen A, Swindle R (2004). Stimulant treatment patterns and compliance in children and adults with newly treated attention-deficit/hyperactivity disorder. J Manag Care Pharm.

[46] Bonvicini C, Faraone SV, Scassellati C (2016). Attention-deficit hyperactivity disorder in adults: a systematic review and meta-analysis of genetic, pharmacogenetic and biochemical studies. Mol Psychiatry.

[47] Polanczyk G, Faraone SV, Bau CH, Victor MM, Becker K, Pelz R, Buitelaar JK, Franke B, Kooij S, van der Meulen E, Cheon KA, Mick E, Purper-Ouakil D, Gorwood P, Stein MA, Cook EH, Rohde LA (2008). The impact of individual and methodological factors in the variability of response to methylphenidate in ADHD pharmacogenetic studies from four different continents. Am J Med Genet B Neuropsychiatr Genet.

[48] Torgersen T, Gjervan B, Nordahl HM, Rasmussen K (2012). Predictive factors for more than 3 years’ duration of central stimulant treatment in adult attention-deficit/hyperactivity disorder: a retrospective, naturalistic study. J Clin Psychopharmacol.

[49] Sobanski E, Retz W, Fischer R, Ose C, Alm B, Hennig O, Rosler M (2014). Treatment adherence and persistence in adult ADHD: results from a 24 week controlled clinical trial with extended release methylphenidate. Eur Psychiatry.

[50] Steele M, Jensen PS, Quinn DM (2006). Remission versus response as the goal of therapy in ADHD: a new standard for the field?. Clin Ther.

[51] Biederman J, Petty CR, Evans M, Small J, Faraone SV (2010). How persistent is ADHD? A controlled 10-year follow-up study of boys with ADHD. Psychiatry Res.

[52] Torgersen T, Gjervan B, Rasmussen K (2008). Treatment of adult ADHD: is current knowledge useful to clinicians?. Neuropsychiatr Dis Treat.

[53] Adler LA, Spencer T, Faraone SV, Kessler RC, Howes MJ, Biederman J, Secnik K (2006). Validity of pilot adult ADHD self-report scale (ASRS) to rate adult ADHD symptoms. Ann Clin Psychiatry.

[54] Rosler M, Retz W, Retz-Junginger P, Thome J, Supprian T, Nissen T, Stieglitz RD, Blocher D, Hengesch G, Trott GE (2004). Tools for the diagnosis of attention-deficit/hyperactivity disorder in adults. Self-rating behaviour questionnaire and diagnostic checklist. Nervenarzt.

[55] Stirratt MJ, Dunbar-Jacob J, Crane HM, Simoni JM, Czajkowski S, Hilliard ME, Aikens JE, Hunter CM, Velligan DI, Huntley K, Ogedegbe G, Rand CS, Schron E, Nilsen WJ (2015). Self-report measures of medication adherence behavior: recommendations on optimal use. Transl Behav Med.

[56] Murphy P, Schachar R (2000). Use of self-ratings in the assessment of symptoms of attention deficit hyperactivity disorder in adults. Am J Psychiatry.

[57] Taylor A, Deb S, Unwin G (2011). Scales for the identification of adults with attention deficit hyperactivity disorder (ADHD): a systematic review. Res Dev Disabil.

[58] Kessler RC, Adler L, Ames M, Demler O, Faraone S, Hiripi E, Howes MJ, Jin R, Secnik K, Spencer T, Ustun TB, Walters EE (2005). The World Health Organization adult ADHD self-report scale (ASRS): a short screening scale for use in the general population. Psychol Med.

